# Outcomes of mechanical thrombectomy in acute stroke patients with atrial fibrillation detected after stroke versus known atrial fibrillation

**DOI:** 10.1007/s11239-023-02923-6

**Published:** 2023-12-21

**Authors:** Lucio D’Anna, Raffaele Ornello, Matteo Foschi, Michele Romoli, Samir Abu-Rumeileh, Tsering Dolkar, Orsolya Vittay, Luke Dixon, Paul Bentley, Zoe Brown, Charles Hall, Sohaa Jamil, Harri Jenkins, Joseph Kwan, Maneesh Patel, Neil Rane, Dylan Roi, Abhinav Singh, Marius Venter, Dheeraj Kalladka, Abid Malik, Omid Halse, Simona Sacco, Soma Banerjee, Kyriakos Lobotesis

**Affiliations:** 1 Department of Stroke and Neuroscience, Charing Cross Hospital, Imperial College London NHS Healthcare Trust, London, UK; 2 Department of Brain Sciences, Imperial College London, London, UK; 3 Department of Biotechnological and Applied Clinical Sciences, University of L’Aquila, L’Aquila, Italy; 4 Neurology and Stroke Unit, Department of Neuroscience, Bufalini Hospital, AUSL Romagna, Cesena, Italy; 5 Department of Neurology, Martin-Luther-University Halle-Wittenberg, Halle (Saale), Germany; 6 Neuroradiology, Department of Imaging, Charing Cross Hospital, Imperial College London, NHS Healthcare Trust, London, UK

**Keywords:** Atrail fibrillation, Mechanical thrombectomy, AFDAS

## Abstract

We aim to compare the outcomes in patients with atrial fibrillation detected after stroke (AFDAS) and their counterparts with known AF (KAF) presenting with large vessel occlusion (LVO) treated with mechanical thrombectomy (MT). This observational, prospective study included consecutive patients with acute LVO ischemic stroke of the anterior circulation with AFDAS, KAF and without AF. The primary study outcome was functional independence at 90 days after stroke. The secondary study outcomes were variation of the NIHSS score at 24 h, rate of successful reperfusion, death at 90 days and rate of immediate complications post-procedure. Overall, our cohort included 518 patients with acute ischemic stroke and LVO treated with MT, with 289 (56.8%) without a diagnosis of AF; 107 (21%) with AFDAS; 122 (22.2%) with KAF. There was no significant difference in terms of functional independence at 90 days after stroke between the three groups. Regarding the secondary study outcome, the rate of symptomatic intracranial haemorrhage (sICH) and/or parenchymal hematoma (PH) were significantly higher in the group of patients without AF (respectively, *P* = 0.030 and  < 0.010). Logistic regression analysis showed that the subtypes of AF were not statistically significantly associated with functional independence at 90 days after stroke and with the likelihood of any ICH. Our results suggest that the subtypes of AF are not associated with clinical and safety outcomes of MT in patients with acute stroke and LVO. Further studies are needed to confirm our findings.

**Supplementary Information:**

The online version contains supplementary material available at 10.1007/s11239-023-02923-6.

## Introduction

Atrial fibrillation (AF) is associated with up to a five-fold increase in stroke risk [1], and the prevalence of AF in patients diagnosed with ischemic stroke varies from 11 to 33% depending on the study design and methods used to detect AF [2–4]. AF can be newly detected in close temporal proximity to the index stroke (AFDAS) or can be known before the index stroke (known or KAF) [5]. Oral anticoagulation (OAC) is recommended by American and European guidelines to reduce the risk of stroke and systemic embolism in patients with AF [6–8]. However, despite the OAC treatment, 1–2% of AF patients suffer from an acute ischemic stroke per year, and about 10% of all ischemic stroke patients have KAF on OAC therapy at stroke onset [9–12].

MT is effective and safe in acute ischemic stroke due to large vessel occlusion (LVO) of the anterior circulation, irrespective of the cause. Previous meta-analyses have produced conflicting results on the post-MT outcomes in acute LVO stroke patients with AF and their counterparts without AF [13, 14]. To date limited evidence is available on the outcome profile between patients with AFDAS and KAF and acute ischemic stroke due to LVO following MT. This distinction may be important in the context of LVO stroke because patients with KAF are more likely to be already on treatment with OAC prior to the stroke and this could influence the burden, location and clot composition and tendency for haemorrhagic transformation [15–19]. Indeed, thrombi retrieved from KAF patients on treatment with OAC contain more fibrins, more platelets and fewer red blood cells; in these patients a trend of higher successful reperfusion rate was observed but failed to reach statistical significance compared to AFDAS patients [15]. Therefore, our study aimed to investigate differences in terms of clinical, reperfusion and safety outcomes of patients with KAF and AFDAS treated with mechanical thrombectomy.

## Patients and methods

### Study design and patients

This is an observational, investigator-initiated, prospective study, that included all acute stroke patients consecutively treated with MT at the Stroke Department, Charing Cross Hospital, Imperial College Healthcare NHS Trust, London between 1st January 2016 and 30th June 2021. The study was conducted in accordance with the recommendations for physicians involved in research on human subjects adopted by the 18th World Medical Assembly, Helsinki 1964 and later revisions. The Stroke Department at Charing Cross Hospital is the Northwest London (UK) regional Comprehensive Stroke Centre (CSC) for MT in an urban metropolitan area with more than 6.4 million people. Please refer to our previous manuscripts for the organization of the Imperial Stroke Thrombectomy network [22, 23].

### Patient inclusion and exclusion criteria for the analysis

For the purpose of this analysis, the criteria for patients selection were: (1) age ≥ 18 years; (2) NIHSS score 6 or more; (3) Alberta Stroke Program Early CT score (ASPECTS) [24] 5 or more; (4) LVO sites: distal internal carotid artery, middle cerebral artery segments M1 or M2; (5) initiation of the MT had to be possible within 6 h after the stroke onset; (6) modified Rankin Scale (mRS) score of 0–2. IVT was administered in all patients who presented within 4.5 h of stroke symptom onset and without contraindications according to the guidelines. For this analysis, we excluded stroke patients with basilar artery occlusion and patients that met DAWN or DEFUSE 3 eligibility criteria [25, 26]. KAF was defined as documented evidence of AF before the index event. AFDAS was defined if found on admission ECG or during the hospital admission.

### Clinical and radiological assessments

Please refer to supplemental materials.

### Outcomes

The primary study outcome was functional independence at 90 days after stroke (defined as mRS scores of 0–2). The secondary study outcomes were variation of the NIHSS score at 24 h, the rate of successful reperfusion (defined by applying the modified thrombolysis in cerebral infarction (TICI) classification [27]; successful recanalization was defined as grade 2b, 2c or 3 of reperfusion), death at 90 days and the rate of immediate complications post-procedure (in hospital death, malignant middle cerebral artery syndrome, hemicraniectomy, rate of haemorrhagic transformations and symptomatic intracranial haemorrhage).

### Statistical analysis

Descriptive categorical data were reported as numbers and proportions; descriptive continuous data were reported as means and standard deviations (SDs) for normally distributed variables, including age and blood pressure values, or medians and interquartile ranges (IQRs) for non-normally distributed variables, including stroke scale scores. We compared the demographic, clinical, and procedure-related characteristics of the three groups (no AF, AFDAS, and known AF) by chi-square test (for categorical variables), one-way ANOVA (for normally distributed continuous variables, followed by Tukey’s post hoc test), or Kruskal–Wallis test (for non-normally distributed continuous variables). P values were considered statistically significant at < 0.05. We performed a univariate logistic regression analysis with calculation of odds ratios (ORs) and 95% confidence intervals (Cis) to investigate variables associated with the study outcomes. Variables with a significant association with the study outcomes (P ≤ 0.1) were considered for multivariate logistic regression analysis with statistical significance set at a P < 0.05. Statistical analyses were performed with R software, version 4.2.2.

## Results

**Fig. 1:**
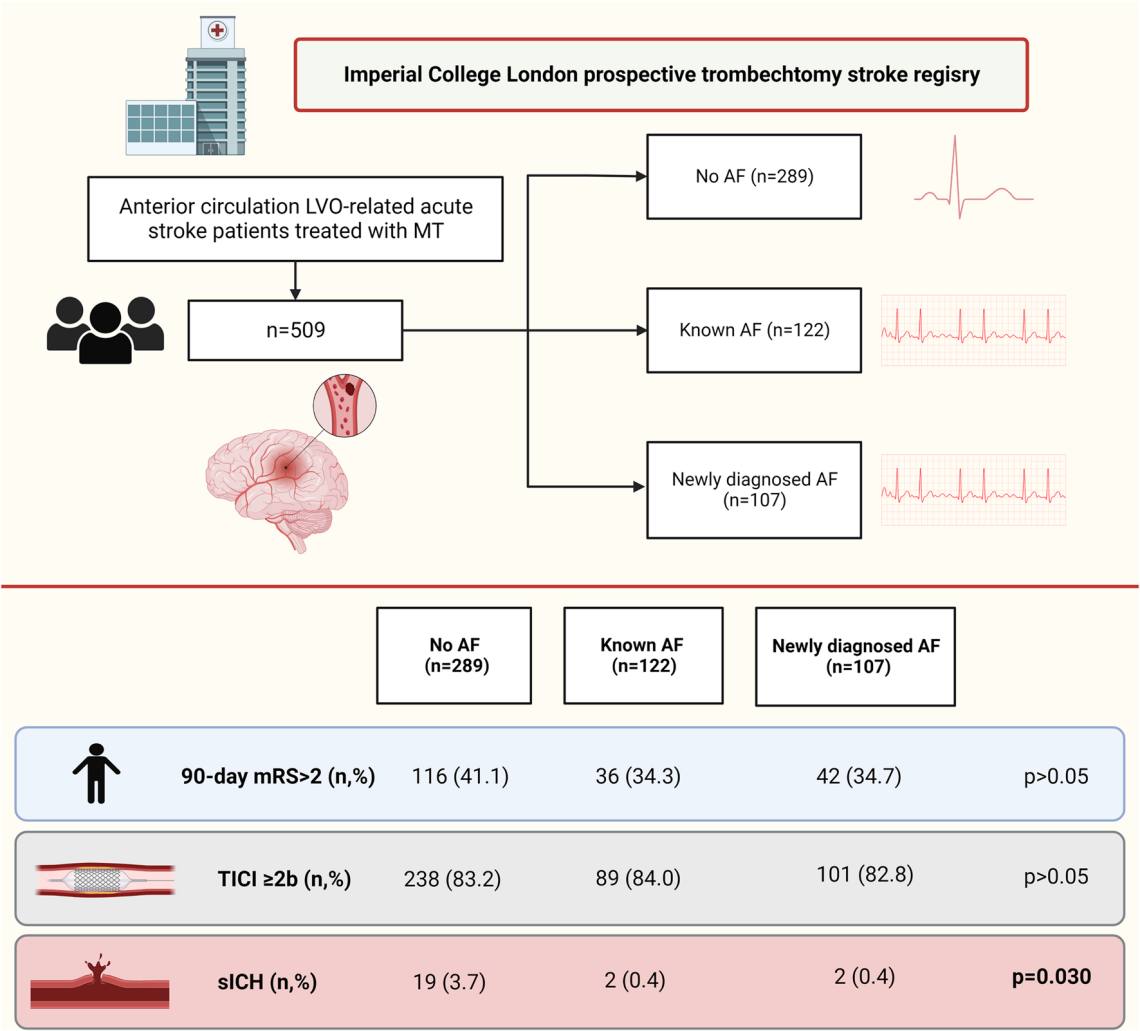
Study algorithm and results

Our analysis included 518 patients with acute ischemic stroke due to LVO treated with MT (Fig. 1). Among the included patients, 289 (56.8%) patients did not have a diagnosis of AF, 107 (21%) patients had AFDAS and 122 (22.2%) patients had KAF. Demographic and clinical features of our sample are reported in Table 1. Patients with no AF were significantly younger (p < 0.001), more frequently male (p = 0.021) and non-smoker (p < 0.001), had a lower prevalence of hypertension (p = 0.001) but a higher rate of symptomatic carotid artery stenosis (p < 0.001) compared to the other two groups. Patients with KAF had a higher rate of previous TIA/ischemic stroke (p < 0.001) and previous intracranial haemorrhage (p = 0.049). We documented that the distribution of the CHAD2DS2 VASC score was significantly different between the three groups (p < 0.001). In terms of admission therapy, patients with KAF were more frequently on anticoagulation (p < 0.001), statins (p = 0.002) and blood pressure-lowering drugs (p < 0.001) compared with the other two groups. Procedural features were reported in Supplemental Table 1. As expected, MT alone was more frequently performed in the group of patients with KAF (p < 0.001). There were no significant differences between the three groups regarding the other variables taken into consideration.

**Table 1:** Demographic and clinical characteristics

	Patients with no atrial fibrillation (n = 289)	Patients with AFDAS (n = 107)	Patients with known atrial fibrillation/atrial flutter (n = 122)	p**-**value
**Demographics**				
Age, years [mean ± standard deviation]	62.7 ± 14.6	72.6 ± 13.4	74.9 ± 9.6	< 0.001*
Female sex [n (%)]	116 (40.1)	50 (46.7)	67 (54.9)	0.021
**Cardiovascular risk factors**				
Hypertension [n (%)]	137 (47.4)	64 (59.8)	82 (67.2)	0.001
Diabetes mellitus [n (%)]	59 (20.4)	17 (15.9)	22 (18.0	0.570
Hypercholesterolemia [n (%)]	120 (41.5)	55 (51.4)	62 (50.8)	0.094
Smoking status [n (%)]				< 0.001
Never smoker	212 (73.4)	94 (87.9)	103 (84.4)	
Current smoking	62 (21.4)	6 (5.6)	9 (7.4)	
Former smoking	15 (5.2)	7 (6.5)	10 (8.2)	
Current alcohol consumption [n (%)]	13 (4.5)	6 (5.6)	2 (1.6)	0.267
Coronary artery disease [n (%)]	45 (15.6)	17 (15.9)	24 (19.7)	0.579
Congestive heart failure [n (%)]	23 (8.0)	9 (8.4)	18 (14.8)	0.092
Symptomatic carotid artery disease [n (%)]	45 (15.6)	2 (1.9)	3 (2.5)	< 0.001
Peripheral artery disease [n (%)]	11 (3.8)	3 (2.8)	10 (8.2)	0.092
Previous TIA/ischemic stroke [n (%)]	45 (15.6)	10 (9.3)	49 (40.2)	< 0.001
Previous history of intracranial hemorrhage [n (%)]	1 (0.4)	–	3 (2.5)	0.049
Dementia [n (%)]	3 (1.0)	–	–	0.303
Malignancy [n (%)]	24 (8.3)	11 (10.3)	15 (12.3)	0.443
Total CHA2DS2 VASC score [median (IQR)]	4 (3–5)	5 (3.5–6)	5 (4–6)	< 0.001
**Admission therapy**				
Anticoagulation on admission [n (%)]	13 (4.5)	2 (1.9)	76 (62.3)	< 0.001
Antiplatelet therapy on admission [n (%)]				0.368
Aspirin	51 (17.8)	13 (12.6)	13 (10.7)	
Clopidogrel	20 (7.0)	6 (5.8)	6 (5.0)	
DAPT	6 (2.1)	3 (2.9)	1 (0.8)	
Statins on admission [n (%)]	84 (29.5)	39 (39.0)	57 (47.1)	0.002
Blood pressure-lowering drugs on admission [n (%)]	118 (41.3)	54 (52.4)	90 (74.4)	< 0.001
NIHSS on admission [median (IQR)]	17 (13–20)	18 (13.5–21)	18 (13–22)	0.073
ASPECTS score [median (IQR)]	8 (7–9)	8 (7–9)	8 (7–9)	0.454
**Site of occlusion [n (%)]**				0.170
ICA	19 (6.6)	4 (3.7)	8 (6.6)	
M1	140 (48.4)	64 (59.8)	71 (58.2)	
M2	41 (14.2)	19 (17.8)	16 (13.1)	
ICA + M1	71 (24.6)	14 (13.1)	19 (15.6)	
M1 + M2	18 (6.2)	6 (5.6)	8 (6.6)	

*ASPECTS* The Alberta Stroke Program Early CT Score, *DAPT* dual antiplatelet therapy, *DOAC* direct oral anticoagulant, *ICA* internal carotid artery, *IQR* interquartile range, *mRS* modified Rankin Scale, *LMWH* low molecular weight heparin, *NIHSS* National Institutes of Health Stroke Scale, AF detected after stroke or transient ischemic attack (AFDAS)

*Bonferroni: p < 0.001 (AFDAS vs no), p < 0.001 (known vs no), p = 0.620 (AFDAS vs known)

### Study endpoints

In terms of primary study outcome there was no significant difference regarding the functional independence at 3 months between the three groups (p = 0.311) (Table 2) (Fig. 1). Regarding the secondary study outcomes, there was no significant difference in the variation of the NIHSS score at 24 h (p = 0.945), mortality at 90 days (p = 0.130) and successful recanalization post-procedure (p = 0.971) between the three groups. In terms of immediate complications post-procedure, the following variables did not differ: in-hospital death, malignant middle cerebral artery, hemicraniectomy, rate of hemorrhagic transformation (HT), parenchymal hemorrhage (PH) and subarachnoid hemorrhage (SAH). Conversely, the rate of symptomatic intracranial hemorrhage (sICH) on follow-up CT at 24 h and the rate of sICH and/or PH were significantly higher in the group of patients without AF (respectively, *P* = 0.030 and < 0.010). Logistic regression analysis showed that the subtypes of AF were not statistically significantly associated with functional independence at 90 days after stroke (Supplemental Table 2) and with the likelihood of any ICH (Supplemental Table 3).

**Table 2:** Primary and secondary study outcomes

	Patients with no atrial fibrillation (n = 289)	Patients with new-onset AFDAS (n = 107)	Patients with known atrial fibrillation/atrial flutter (n = 122)	p-value
**Primary study outcome **				
90-day mRS [n (%)]				0.311
≤ 2	173 (59.9)	71 (66.4)	80 (65.6)	
> 2	116 (40.1)	36 (33.6)	42 (34.4)	
**Secondary study outcome**				
∆ NIHSS 24 h [median, (IQR)]	11 (5–19)	12 (6–21)	12 (4–23)	0.945
Post-intervention TICI [n (%)]				0.971
Unfavorable (0, 1, 2a)	48 (16.8)	17 (16.0)	21 (17.2)	
Favorable (2b, 2c, 3)	238 (83.2)	89 (84.0)	101 (82.8)	
Death at 90 days [n (%)]	28 (9.7)	5 (4.7)	15 (12.3)	0.130
Immediate complications post-procedure				
In hospital death [n (%)]	28 (9.7)	5 (4.7)	15 (12.3)	0.177
Malignant MCA infarction [n (%)]	35 (12.1)	11 (10.4)	11 (9.0)	0.639
Hemicraniectomy [n (%)]	14 (4.8)	3 (2.8)	3 (2.5)	0.428
HT [n (%)]	72 (24.9)	22 (20.6)	30 (24.6)	0.654
HT score [n (%)]				0.176
1a	17 (28.8)	4 (23.5)	4 (16.7)	
1b	14 (23.7)	10 (58.8)	12 (50.0)	
1c	3 (5.1)	2 (11.8)	1 (4.2)	
2	9 (15.3)	1 (5.9)	3 (12.5)	
3a	3 (5.1)	–	–	
3b	3 (5.1)	–	–	
3c	10 (16.9)	–	4 (16.7)	
PH (HT 1c, 2, 3,a 3ab)	27 (10.1)	4 (3.9)	9 (7.5)	0.206
SAH (HT 3c) [n (%)]	10 (3.5)	1 (1.0)	–	0.053
sICH [n (%)]	19 (3.7)	2 (0.4)	2 (0.4)	0.030
sICH and/or PH	46 (15.9)	6 (5.6)	11 (9.0)	0.010

*HT* hemorrhagic transformation, *MCA* middle cerebral artery, *PH* parenchymal hemorrhage, *SAH* subarachnoid hemorrhage, *sICH* symptomatic intracranial hemorrhage, *mRS* modified Rankin Scale, AF detected after stroke or transient ischemic attack (AFDAS)

## Discussion

In our cohort of patients with acute LVO ischemic stroke treated with MT, we did not observe a significant difference in terms of functional independence at 3 months when we compared patients with KAF to patients with AFDAS and their counterparts without AF. Recent evidence suggested that that AF detected after ischemic stroke might have a different pathophysiology compared to KAF [28]. In detail, KAF is most likely caused by cardiac structural changes and thus, the arrhythmia could be considered to have a primarily ‘cardiogenic’ pathophysiology [28]. AFDAS may, on the other hand, also be the consequence of the stroke itself and therefore could be regarded as primarily ‘neurogenic’ with involvement of autonomic and inflammatory pathways [20, 21, 29]. More likely, however, a considerable proportion of AF detected after stroke might be related to a combination of cardiogenic and neurogenic mechanisms. Furthermore, clinical factors may differ between patients with AF detected in the setting of acute care or acute illness compared to those with KAF [30]. AFDAS is more often diagnosed in younger and healthier stroke patients whereas KAF remains an independent predictor of mortality after stroke even after adjusting for confounding factors [31]. However, to date the effects of KAF and AFDAS on stroke severity and recurrence in patients with acute ischemic stroke treated with MT [28] are not well understood. To the authors’ knowledge there is only one previous single study in the literature about the role of the different subtypes of AF on outcomes of acute stroke patients treated with MT. Here, Leker et al. investigated the influence of AF temporal detection on outcome after MT [32] and found no significant impact of the different subtypes of AF diagnosis on favourable mRS outcome following MT. Our data confirms these preliminary results in a larger cohort of patients with significant prognostic implications for the management of acute ischemic stroke patients presenting with LVO.

Another important finding of our study is that the subtypes of AF were not statistically significantly associated, in the adjusted model, with an increased likelihood of ICH following MT in patients with acute ischemic stroke. In our analysis 62.3% of the KAF patients were on treatment with anticoagulants before the index event. Interestingly, we did not observe an increased rate of sICH or any HT in patients with KAF as might have been expected. The unadjusted analysis showed higher rates of sICH in patients with no-AF that could be explained considering differences in the use of systemic thrombolysis. Previous studies documented that therapeutic anticoagulation may provide benefits in terms of short and long-term outcomes, survival, and functional recovery in patients with AF-related stroke [33, 34]. However, these studies did not focus solely on patients with acute stroke due to LVO but on all stroke subtypes also including those with distal branch occlusions. Indeed, these studies recruited patients with an average NIHSS score on admission that was lower than the average score reported in our analysis. Thus, it is certainly possible that in patients with smaller strokes there might be a benefit from prior treatment with oral anticoagulants, though this is not well proven in the subgroup of patients with acute ischemic stroke and LVO. It is noteworthy to mention that AF per se is not a risk factor for sICH, as suggested by previous studies [35, 36]. Additionally, a meta-analysis suggested that AF is a risk factor for sICH after intravenous thrombolysis, but not after MT [37]. In our study population, the low proportion of patients treated with intravenous thrombolysis might explain the low rate of sICH in patients with KAF. Previous data suggest that LVO due to large-artery atherosclerosis or cardioembolism have similar outcomes following MT. In our analysis we did not include variables like collateral vessel status, low lesion volume, and low number of MT passes that could explain the low rate of sICH in patients with new-onset AF; however, collateral status was reported as more favourable in large-artery atherosclerosis than in AF-related stroke.

Our analysis had the following strengths: (1) data ascertainment was undertaken systematically and prospectively; (2) large cohorts of patients as a single centre study. Nevertheless, our study has several limitations. The non-randomized design is likely to have introduced biases. The results could be influenced by numerous potential confounders, even if statistical models were used to adjust for them. However, in several circumstances in which randomized trials are not available, observational studies are considered a useful tool to understand the effects of a treatment or of different clinical services. In addition, if rigorously designed, this type of analysis could help to estimate the effects of interventions, particularly in everyday clinical practice [38]. Despite our efforts, we cannot exclude that our results could have been influenced by an incomplete adjustment for patient characteristics in selecting the model for the analysis. AFDAS is atrial fibrillation detected in close temporal proximity to the index stroke with an admission ECG or during the hospital admission. We can assume that, as this AF has never been recorded before, it happened prior to the event but it is also possible that some of the newly diagnosed AF were actually already present but unrecognized. Nevertheless, those patients, being unaware of the condition, did not receive any specific treatment or advice (only 2% of patients of this group were on oral anticoagulation but the indication was not AF).

In this purely observational study employing a convenience sample, we were unable to compute an a priori effect size. However, post hoc, we calculated statistical power based on the prevalence of the primary outcome, which was defined as a 90-day modified Rankin Scale score of 0–2. Among the patient groups, 119 out of 289 (41.2%) patients without atrial fibrillation (AF), 36 out of 107 (33.6%) AFDAS patients, and 42 out of 122 (34.4%) patients with KAF achieved this outcome. Using these figures, we estimated the minimum sample size required for a chi-squared test using G*Power (version 3.1). Our calculations yielded an effect size of w = 0.485, indicating that a minimum of 70 subjects are needed to achieve a 90% statistical power for the primary outcome. Finally, in our hospital it was not possible to test the direct oral anticoagulant level in the blood.

## Conclusion

The findings of our study suggest that the subtypes of AF studied are not associated with clinical and safety outcomes in patients with acute ischemic stroke and LVO undergoing MT. Further studies in larger samples are needed to confirm our findings.

### Supplementary Information

Below is the link to the electronic supplementary material.

Supplementary file1 (DOCX 57 KB)

Supplementary file2 (DOCX 42 KB)

## Data Availability

Data available upon reasonable request.
