# Supplementary material for: Outcomes of mechanical thrombectomy in acute stroke patients with atrial fibrillation detected after stroke versus known atrial fibrillation
Source: J Thromb Thrombolysis. 2023 Dec 21;57(3):445–52. doi: 10.1007/s11239-023-02923-6 (PMC10961279; doi:10.1007/s11239-023-02923-6)
Supplement: Supplementary file 1 — Supplementary file1 (DOCX 57 KB) [file 11239_2023_2923_MOESM1_ESM.docx]

**Supplemental Table 1. Procedural features.**

|  | **Patients with no atrial fibrillation**  **(n = 289)** | **Patients with new-onset Atrial Fibrillation/Atrial Flutter**  **(n = 107)** | **Patients with known Atrial Fibrillation/Atrial Flutter**  **(n = 122)** | **p-value** |
| --- | --- | --- | --- | --- |
| Delivery to the CSC, n (%)  MS  DS | 108 (37.4)  181 (62.6) | 47 (43.9)  60 (56.1) | 38 (31.1)  84 (68.9) | 0.136 |
|  |  |  |  |  |
| Type of procedure, n (%)  IVT+MT  MT alone | 233 (80.6)  56 (19.4) | 89 (83.2)  18 (16.8) | 46 (37.7)  76 (62.3) | **<0.001** |
| Type of anesthesia, n (%) |  |  |  | 0.213 |
| GA | 180 (62.3) | 66 (62.3) | 75 (61.5) |  |
| LA | 103 (35.6) | 40 (37.7) | 47 (38.5) |  |
| Converted LA to GA | 6 (2.1) | - | - |  |
| Onset to needle time (minutes) [mean ± standard deviation] | 124 (92-160) | 120 (94-155) | 135 (95-161) | 0.958 |
| Thrombolytic agent, n (%) |  |  |  | 0.353 |
| Alteplase, n (%) | 223 (96.1) | 83 (95.4) | 47 (100.0) |  |
| Tenecteplase, n (%) | 9 (3.9) | 4 (4.6) | - |  |
|  |  |  |  |  |
| Onset to groin puncture time (minutes) [mean ± standard deviation] | 280 (228-324) | 281 (221-343) | 290 (240-331) | 0.516 |
| Thromboaspiration [n, (%)] | 177 (61.9) | 71 (66.4) | 82 (67.2) | 0.507 |
| Stent retriever [n, (%)] | 140 (48.6) | 47 (43.9) | 57 (46.7) | 0.704 |
| Combination of aspiration/stent retriever [n, (%)] | 78 (27.0) | 26 (24.3) | 37 (30.3) | 0.501 |
| Pre-intervention TICI [n, (%)] |  |  |  | 0.954 |
| 0 | 250 (87.1) | 90 (85.7) | 109 (89.3) |  |
| 1 | 15 (5.2) | 4 (3.8) | 5 (4.1) |  |
| 2a | 11 (3.8) | 6 (5.7) | 4 (3.3) |  |
| 2b | 10 (3.5) | 5 (4.8) | 4 (3.3) |  |
| 3 | 1 (0.3) | - | - |  |
|  |  |  |  |  |

**Legend:** CSC = comprehensive stroke center; DS = drip and ship; GA = general anesthesia; IQR = interquartile range; IVT = intravenous thrombolysis; LA = local anesthesia; MS = mothership; MT = mechanical thrombectomy; TICI = Thrombolysis in cerebral infarction

**Supplemental Table 2. Predictors of mRS >2: univariate and multivariate regression analysis**

|  | **Univariate regression OR (95% CI)** | **p value** | **Multivariate regression OR (95% CI)** | **p value** |
| --- | --- | --- | --- | --- |
| *Demographics* |  |  |  |  |
| Age, per year | 1.01 (1.00-1.01) | <0.001 | 1.00 (0.98-1.01) | 0.719 |
| Male gender | 0.94 (0.86-1.02) | 0.137 | - |  |
| *Medical history* |  |  |  |  |
| Atrial fibrillation |  |  |  |  |
| No | 1.00 (Ref.) |  | - |  |
| Known | 1.07 (0.96-1.19) | 0.218 | - |  |
| New-onset | 1.07 (0.96-1.18) | 0.225 | - |  |
| Diabetes | 1.09 (0.98-1.22) | 0.102 | - |  |
| Hypercholesterolaemia | 0.90 (0.83-0.98) | 0.017 | 0.53 (0.33-0.85) | 0.015 |
| Coronary artery disease | 1.08 (0.96-1.20) | 0.213 | - |  |
| Symptomatic carotid artery disease | 0.98 (0.85-1.13) | 0.782 | - |  |
| Congestive heart failure | 1.02 (0.88-1.17) | 0.826 | - |  |
| Vascular disease | 1.01 (0.83-1.23) | 0.943 | - |  |
| Smoking | 1.04 (0.85-1.27) | 0.693 | - |  |
| Alcohol abuse | 0.63 (0.32-1.26) | 0.192 | - |  |
| History of Drug use | 1.05 (0.71-1.55) | 0.806 | - |  |
| Previous history of ischaemic stroke | 1.12 (0.99-1.28) | 0.077 | 0.57 (0.33-1.00) | 0.063 |
| Previous history of transient ischaemic attack | 0.97 (0.83-1.13) | 0.653 | - |  |
| Previous history of intracranial haemorrhage | 1.14 (0.71-1.84) | 0.587 | - |  |
| History of malignancy | 1.02 (0.89-1.18) | 0.738 | - |  |
| History of dementia | 1.05 (0.60-1.82) | 0.863 | - |  |
| Modified Rankin Scale (mRS) score pre-stroke, per point increase | 1.16 (1.09-1.24) | <0.001 | 1.07 (0.84-1.37) | 0.573 |
| Total CHA2DS2-VASc score, per point increase | 1.05 (1.02-1.08) | <0.001 | 1.01 (0.87-1.17) | 0.897 |
| Total HAS-BLED score, per point increase | 1.07 (1.03-1.12) | 0.002 | 0.99 (0.84-1.16) | 0.900 |
| *Medication prior to the index event* |  |  |  |  |
| Anticoagulants | 1.07 (0.96-1.91) | 0.252 | - |  |
| Antiplatelet agents prior to the index event |  |  |  |  |
| ASA | 1.00 (Ref.) |  |  |  |
| Clopidogrel | 0.90 (0.73-1.10) | 0.310 |  |  |
| DAPT | 0.90 (0.65-1.24) | 0.515 |  |  |
| None | 0.90 (0.80-1.02) | 0.102 |  |  |
| Antihypertensives | 1.12 (1.04-1.22) | 0.008 | 0.81 (0.55-1.19) | 0.291 |
| Statins | 1.11 (1.01-1.21) | 0.028 | 1.55 (0.91-2.63) | 0.123 |
| *Pre-hospital variables* |  |  |  |  |
| Thrombolysis | 0.87 (0.80-0.96) | 0.004 | 1.01 (0.64-1.58) | 0.972 |
| Centralisation to comprehensive stroke centre | 1.05 (0.96-1.14) | 0.295 | - |  |
| Large vessel occlusion | 1.13 (0.84-3.24) | 0.723 | - |  |
| Anaesthesia used for thrombectomy |  |  |  |  |
| Conscious sedation | 1.00 (Ref.) |  |  |  |
| Local anaesthesia | 1.07 (0.72-1.59) | 0.723 | - |  |
| General anaesthesia | 1.15 (0.78-1.70) | 0.487 | - |  |
| NIHSS score on presentation | 1.02 (1.01-1.03) | <0.001 | 1.01 (0.99-1.04) | 0.372 |
| NIHSS score at 24 hour | 1.02 (1.02-1.02) | <0.001 | 0.99 (0.98-1.01) | 0.492 |
| Onset-to-door time, per minute increase | 1.00 (0.99-1.00) | 0.419 | - |  |
| Onset-to-needle time, per minute increase (patients treated with intravenous thrombolysis) | 1.00 (0.99-1.00) | 0.387 | - |  |
| Door-to-needle time, per minute increase (patients treated with intravenous thrombolysis) | 1.00 (0.99-1.00) | 0.733 | - |  |
| Onset-to-groin puncture time, per minute increase | 1.00 (0.99-1.00) | 0.184 | - |  |
| Door-to-groin puncture time, per minute increase | 1.00 (0.99-1.00) | 0.115 | - |  |
| Transport time (for external patients), per minute increase | 1.00 (0.99-1.00) | 0.866 | - |  |
| *Laboratory paramenters* |  |  |  |  |
| Hemoglobin (g/dL), per unit increase | 0.99 (0.99-0.99) | 0.002 | 1.00 (0.99-1.00) | 0.178 |
| Platelets (10^9/L), per unit increase | 0.99 (0.99-1.00) | 0.129 | - |  |
| Activated prothrombin time (secs), per unit increase | 1.00 (0.99-1.00) | 0.554 | - |  |
| International Normalised Ratio, per unit increase | 1.00 (0.99-1.01) | 0.203 | - |  |
| Potassium (mmol/L), per unit increase | 1.00 (0.99-1.02) | 0.574 | - |  |
| Sodium (mmol/L), per unit increase | 1.00 (0.99-1.01) | 0.756 | - |  |
| Creatinine (umol/L), per unit increase | 1.00 (0.99-1.00) | 0.161 | - |  |
| Urea (mmol/L), per unit increase | 1.00 (0.99-1.00) | 0.560 | - |  |
| Estimated glomerular filtration rate (mL/min/1.73m^2^), per unit increase | 1.00 (0.99-1.00) | 0.003 | 0.99 (0.99-1.00) | 0.339 |
| Alanine aminotransferase ALT (U/L) , per unit increase | 1.00 (1.00-1.00) | 0.527 | - |  |
| Alkaline phosphatase (U/L), per unit increase | 1.00 (1.00-1.00) | 0.156 | - |  |
| Bilirubin (µmol/L) | 1.00 (1.00-1.00) | 0.706 | - |  |
| *Vital signs* |  |  |  |  |
| Systolic blood pressure at admission (mmHg), per unit increase | 1.00 (1.00-1.00) | 0.023 | 1.00 (0.99-1.01) | 0.243 |
| Diastolic blood pressure at admission (mmHg), per unit increase | 1.00 (1.00-1.00) | 0.735 | - |  |
| Heart rate (beats/minute), per unit increase | 1.00 (1.00-1.00) | 0.945 | - |  |
| *Procedure-related variables* |  |  |  |  |
| ASPECT score | 0.95 (0.92-0.98) | 0.001 | 0.97 (0.88-1.06) | 0.458 |
| Cervical carotid stenting | 2.02 (0.83-1.23) | 0.942 | - |  |
| Cervical carotid angioplasty | 1.09 (0.88-1.36) | 0.438 | - |  |
| First pass aspiration technique used? | 0.97 (0.81-1.16) | 0.720 | - |  |
| First pass aspiration technique successful? | 0.86 (0.70-1.05) | 0.133 | - |  |
| Thrombo-aspiration system | 1.02 (0.93-1.11) | 0.692 | - |  |
| Stent retriever | 1.14 (1.04-1.24) | 0.003 | 0.85 (0.60-1.20) | 0.361 |
| Combination of aspiration/retriever/ Device used | 0.72 (0.28-1.87) | 0.499 | - |  |
| Proximal balloon/flow arrest guide catheter | 0.99 (0.85-1.15) | 0.897 | - |  |
| Distal catheter access | 1.04 (0.93-1.17) | 0.491 | - |  |
| Distal clot migration or embolization | 0.68 (0.55-0.86) | 0.001 | 0.68 (0.47-0.97) | 0.046 |
| Pre-intervention TICI score |  |  | - |  |
| 0 | 1.00 (Ref.) |  |  |  |
| 1 | 0.97 (0.79-1.19) | 0.769 |  |  |
| 2a | 1.03 (0.83-1.28) | 0.803 |  |  |
| 2b | 0.91 (0.73-1.14) | 0.402 |  |  |
| 3 | 1.46 (0.56-3.80) | 0.439 |  |  |
| Post-intervention TICI score |  |  |  |  |
| 0 | 1.00 (Ref.) |  |  |  |
| 1 | 1.17 (0.84-1.63) | 0.351 |  |  |
| 2a | 0.93 (0.75-1.16) | 0.527 |  |  |
| 2b | 0.81 (0.69-0.96) | 0.017 | 1.13 (0.41-3.10) | 0.810 |
| 2c | 0.77 (0.63-0.95) | 0.017 | 0.69 (0.41-1.17) | 0.184 |
| 3 | 0.73 (0.62-0.86) | <0.001 | 0.68 (0.41-1.15) | 0.164 |
| Ward of admission after treatment |  |  | - |  |
| Stroke care unit | 1.00 (Ref.) |  |  |  |
| Intensive care unit | 0.80 (0.31-2.08) | 0.652 |  |  |
| Malignant middle cerebral artery infarction | 1.34 (1.17-1.53) | <0.001 | 1.30 (0.52-3.28) | 0.581 |
| Hemicraniectomy | 1.33 (1.07-1.67) | 0.011 | 0.77 (0.94-15.51) | 0.710 |
| Haemorrhagic transformation within 24 hours | 1.16 (1.05-1.29) | 0.003 | 2.54 (1.36-4.74) | 0.008 |
| Haemorrhagic transformation within 48-72 hrs (Highest score) |  |  |  |  |
| 1a | 1.00 (Ref.) |  |  |  |
| 1b | 0.95 (0.67-1.36) | 0.784 |  |  |
| 1c | 1.28 (0.76-2.16) | 0.352 |  |  |
| 2 | 0.63 (0.41-0.98) | 0.045 | Not estimable | - |
| 3a | 1.28 (0.52-3.16) | 0.590 |  |  |
| 3c | 1.28 (0.76-2.16) | 0.352 |  |  |
| Haemorrhagic infarction | 1.10 (0.99-1.21) | 0.063 | 0.45 (1.71-2.88) | 0.011 |
| Parenchymal haematoma | 1.01 (0.86-1.18) | 0.926 | - |  |
| Subarachnoid haemorrhage | 1.20 (0.89-1.63) | 0.233 | - |  |
| Symptomatic intracranial haemorrhage (i.e. ≥4-point increase in NIHSS score) | 1.42 (1.16-1.75) | 0.001 | 0.26 (0.05-1.37) | 0.127 |
| Drip-and-ship | 1.05 (0.96-1.14) | 0.295 | - |  |

**Supplemental Table 3. Predictors of symptomatic intracranial hemorrhage: univariate and multivariate regression analyses**

|  | **Univariate regression OR (95% CI)** | **P value** | **Multivariate regression OR (95% CI)** | **P value** |
| --- | --- | --- | --- | --- |
| *Demographics* |  |  |  |  |
| Age at the time of event | 1.00 (1.00-1.00) | 0.801 | - | - |
| Gender MAle | 1.02 (0.97-1.07) | 0.404 | - | - |
| *Medical history* |  |  |  |  |
| Atrial fibrillation |  |  |  |  |
| No | 1.00 (Ref.) |  |  |  |
| Known | 0.95 (0.89-1.00) | 0.064 | 1.04 (0.89-1.22) | 0.599 |
| New-onset | 0.98 (0.93-1.04) | 0.495 | 1.14 (0.98-1.32) | 0.096 |
| Diabetes | 1.08 (0.96-1.07) | 0.643 | - | - |
| Hypercholesterolaemia | 1.03 (0.98-1.08) | 0.222 | - | - |
| Coronary artery disease | 1.08 (1.01-1.15) | 0.018 | 0.92 (0.79-1.08) | 0.313 |
| Symptomatic carotid artery disease | 0.96 (0.89-1.11) | 0.301 | - | - |
| Congestive heart failure | 0.94 (0.87-1.01) | 0.111 | - | - |
| Vascular disease | 0.96 (0.86-1.07) | 0.505 | - | - |
| Smoking | 1.05 (0.94-1.17) | 0.407 | - | - |
| Alcohol abuse | 1.06 (0.73-1.55) | 0.759 | - | - |
| History of Drug use | 0.92 (0.75-1.13) | 0.442 | - | - |
| Previous history of ischaemic stroke | 1.00 (0.93-1.08) | 0.946 | - | - |
| Previous history of transient ischaemic attack | 1.02 (0.94.1.11) | 0.612 | - | - |
| Previous history of intracranial haemorrhage | 1.19 (0.91-1.55) | 0.194 | - | - |
| History of malignancy | 0.96 (0.89-1.04) | 0.301 | - | - |
| History of dementia | 1.29 (0.96-1.75) | 0.096 | Not estimable | - |
| Modified Rankin Scale (mRS) score pre-stroke, per point increase | 0.99 (0.96-1.03) | 0.730 | - | - |
| Total CHA2DS2-VASc score, per point increase | 1.00 (0.98-1.01) | 0.963 | - | - |
| Total HAS-BLED score, per point increase | 1.01 (0.99-1.04) | 0.392 | - | - |
| *Medication prior to the index event* |  |  |  |  |
| Anticoagulants | 0.96 (0.90-1.02) | 0.189 | - | - |
| Antiplatelet agents prior to the index event |  |  |  |  |
| ASA | 1.00 (Ref.) |  |  |  |
| Clopidogrel | 0.96 (0.86-1.07) | 0.522 | 1.10 (0.84-1.43) | 0.504 |
| DAPT | 1.07 (0.90-1.28) | 0.437 | 1.45 (1.02-2.07) | 0.042 |
| None | 0.94 (0.88-1.00) | 0.049 | 0.97 (0.81-1.16) | 0.714 |
| Antihypertensives | 1.02 (0.97-1.07) | 0.420 | - |  |
| Statins | 1.05 (1.00-1.10) | 0.072 | 1.09 (0.95-1.25) | 0.192 |
| *Pre-hospital variables* |  |  |  |  |
| Thrombolysis | 1.04 (0.99-1.10) | 0.097 | 1.12 (0.98-1.28) | 0.089 |
| Centralisation to comprehensive stroke centre | 1.03 (0.98-1.08) | 0.293 | - | - |
| Large vessel occlusion | 1.08 (0.75-1.57) | 0.682 | - | - |
| Anaesthesia used for thrombectomy |  |  |  |  |
| Conscious sedation | 1.00 (Ref.) |  |  |  |
| Local anaesthesia | 1.07 (0.86-1.32) | 0.567 | - | - |
| General anaesthesia | 1.09 (0.88-1.36) | 0.413 | - | - |
| NIHSS score on presentation | 1.00 (1.00-1.01) | 0.190 | - | - |
| NIHSS score at 24 hour | 1.01 (1.00-1.01) | <0.001 | 1.01 (1.00-1.01) | 0.029 |
| Onset-to-door time, per minute increase | 1.00 (1.00-1.00) | 0.216 | - | - |
| Onset-to-needle time, per minute increase (patients treated with intravenous thrombolysis) | 1.00 (1.00-1.00) | 0.243 | - | - |
| Door-to-needle time, per minute increase (patients treated with intravenous thrombolysis) | 1.00 (1.00-1.00) | 0.120 | - | - |
| Onset-to-groin puncture time, per minute increase | 1.00 (1.00-1.00) | 0.896 | - | - |
| Door-to-groin puncture time, per minute increase | 1.00 (1.00-1.00) | 0.433 | - | - |
| Transport time (for external patients) | 1.00 (1.00-1.00) | 0.525 | - | - |
| *Laboratory parameters* |  |  |  |  |
| Hemoglobin (g/dL), per unit increase | 1.00 (1.00-1.00) | 0.162 | - | - |
| Platelets (10^9/L), per unit increase | 1.00 (1.00-1.00) | 0.271 | - | - |
| Activated prothrombin time (secs), per unit increase | 1.00 (1.00-1.00) | 0.832 | - | - |
| International Normalised Ratio, per unit increase | 1.00 (1.00-1.00) | 0.632 | - | - |
| Potassium (mmol/L), per unit increase | 1.00 (0.99-1.01) | 0.530 | - | - |
| Sodium (mmol/L), per unit increase | 1.00 (1.00-1.00) | 0.533 | - | - |
| Creatinine (umol/L), per unit increase | 1.00 (1.00-1.00) | 0.687 | - | - |
| Urea (mmol/L), per unit increase | 1.00 (1.00-1.00) | 0.781 | - | - |
| Estimated glomerular filtration rate (mL/min/1.73m^2^), per unit increase | 1.00 (1.00-1.00) | 0.542 | - | - |
| Alanine aminotransferase ALT (U/L) , per unit increase | 1.00 (1.00-1.00) | 0.001 | 1.00 (1.00-1.00) | 0.787 |
| Alkaline phosphatase (U/L), per unit increase | 1.00 (1.00-1.00) | 0.842 | - | - |
| Bilirubin (µmol/L) | 1.00 (1.00-1.00) | 0.319 | - | - |
| *Vital signs* |  |  |  |  |
| Systolic blood pressure at admission (mmHg), per unit increase | 1.00 (1.00-1.00) | 0.853 | - | - |
| Diastolic blood pressure at admission (mmHg), per unit increase | 1.00 (1.00-1.00) | 0.253 | - | - |
| Heart rate (beats/minute), per unit increase | 1.00 (1.00-1.00) | 0.836 | - | - |
| *Procedure-related variables* |  |  |  |  |
| ASPECT score | 0.98 (0.96-0.99) | 0.003 | 1.00 (0.96-1.04) | 0.922 |
| Cervical carotid stenting | 1.01 (0.90-1.12) | 0.897 | - | - |
| Cervical carotid angioplasty | 1.08 (0.96-1.22) | 0.209 | - | - |
| First pass aspiration technique used? | 0.97 (0.87-1.08) | 0.567 | - | - |
| First pass aspiration technique successful? | 0.89 (0.79-1.01) | 0.067 | 0.90 (0.77-1.05) | 0.178 |
| Thrombo-aspiration system | 1.05 (1.00-1.10) | 0.037 | 1.00 (0.89-1.13) | 0.959 |
| Stent retriever | 1.05 (1.00-1.10) | 0.044 | 1.02 (0.89-1.19) | 0.745 |
| Combination of aspiration/retriever/ Device used | 1.15 (0.68-1.95) | 0.600 | - | - |
| Proximal balloon/flow arrest guide catheter | 1.01 (0.93-1.09) | 0.809 | - | - |
| Distal catheter access | 1.01 (0.95-1.07) | 0.855 | - | - |
| Distal clot migration or embolization | 1.10 (0.97-1.25) | 0.154 | - | - |
| Pre-intervention TICI score |  |  |  |  |
| 0 | 1.00 (Ref.) |  |  |  |
| 1 | 0.96 (0.86-1.08) | 0.520 | - | - |
| 2a | 1.07 (0.95-1.20) | 0.280 | - | - |
| 2b | 0.97 (0.86-1.10) | 0.688 | - | - |
| 3 | 0.93 (0.55-1.57) | 0.772 | - | - |
| Post-intervention TICI score |  |  |  |  |
| 0 | 1.00 (Ref.) |  |  |  |
| 1 | 0.97 (0.82-1.16) | 0.778 | 0.95 (0.55-1.63) | 0.851 |
| 2a | 1.15 (1.02-1.30) | 0.022 | 1.27 (0.85-1.89) | 0.247 |
| 2b | 1.09 (0.99-1.19) | 0.076 | 1.48 (1.05-2.07) | 0.026 |
| 2c | 1.13 (1.00-1.27) | 0.043 | 1.31 (0.93-1.85) | 0.128 |
| 3 | 1.01 (0.93-1.11) | 0.749 | 1.30 (0.93-1.81) | 0.127 |
| Ward of admission after treatment |  |  |  |  |
| Stroke care unit | 1.00 (Ref.) |  |  |  |
| Intensive care unit | 1.20 (0.71-2.01) | 0.493 | - | - |
| Malignant middle cerebral artery infarction | 1.18 (1.10-1.27) | <0.001 | 1.10 (0.85-1.44) | 0.474 |
| Hemicraniectomy | 1.26 (1.12-1.42) | <0.001 | 1.02 (0.68-1.53) | 0.920 |
| Haemorrhagic transformation within 24 hours | 1.03 (0.98-1.08) | 0.293 | - | - |
