# Supplementary material for: Outcomes of mechanical thrombectomy in acute stroke patients with atrial fibrillation detected after stroke versus known atrial fibrillation
Source: J Thromb Thrombolysis. 2023 Dec 21;57(3):445–52. doi: 10.1007/s11239-023-02923-6 (PMC10961279; doi:10.1007/s11239-023-02923-6)
Supplement: Supplementary file 2 — Supplementary file2 (DOCX 42 KB) [file 11239_2023_2923_MOESM2_ESM.docx]

**Supplemental methods**

*Clinical and Radiological Assessments*

The Imperial Stroke Centre registry prospectively collected data of consecutive patients treated with MT and encompassed patient characteristics, including age, vascular risk factors, laboratory results, relevant medical history including evidence of KAF or newly diagnosed AF. The prescription of any anticoagulant before admission was recorded and included the use of any Direct oral anticoagulant (DOAC) therapy (defined as one of the following drugs and dosages: apixaban 2.5 mg or 5 mg twice daily; dabigatran 110 mg or 150 mg twice daily; edoxaban 30 mg or 60 mg once daily; or rivaroxaban 15 mg or 20 mg once daily).; Vitamin K antagonist (VKA) (defined as treatment with acenocoumarol/ warfarin). The choice of treatment was decided by the treating physician as part of routine clinical care pre-admission. NIHSS was performed in all patients on admission and 24h after the MT. The modified Rankin Scale (mRS) was used to assess the patient’s initial pre-stroke status and the level of functional independence at 90 days of the patients was evaluated centrally through a telemedicine consultation or in-person consultation. Procedural metrics were collected prospectively. The extent of the initial core infarct was determined on pre-therapeutic CT using ASPECTS^1^. In addition, an independent rater (consultant neuroradiologist) who did not participate in the endovascular stroke treatment of included patients, evaluated pre-therapeutic CT, and follow-up CT at 24h. Revascularization was assessed by applying the modified thrombolysis in cerebral infarction (TICI) classification^2^. Successful recanalization was defined as grade 2b, 2c or 3 of reperfusion. Immediate complications post procedure (death at CSC, malignant middle cerebral artery stroke, and haemorrhagic transformation (HT)) were prospectively documented. Haemorrhagic transformation (HT) was defined on follow-up CT at 24 h small petechiae along the margins of the infarct (HI-1) or as more confluent petechiae within the infarcted area but without space-occupying effect (HI-2). Parenchymal haematoma (PH) was defined as haematoma in <30% of the infarcted area with some slight space-occupying effect (PH-1) or as dense haematoma in ≥30% of the infarcted area with substantial space-occupying effect or as any haemorrhagic lesion outside the infarcted area (PH-2). In the case of more than one haemorrhagic lesion on brain scan, the worst possible category was assumed. HT was considered symptomatic if it was not seen on the admission brain scan and there was, subsequently, a suspicion of hemorrhage or a decline in neurological status (an increase of more than 4 points in the NIHSS). In addition, subarachnoid hemorrhages were reported ^3,4^.

1. Pexman JHW, Barber PA, Hill MD, et al. Use of the Alberta Stroke Program Early CT Score (ASPECTS) for assessing CT scans in patients with acute stroke. *American Journal of Neuroradiology* 2001; 22: 1534–1542.

2. Higashida RT, Furlan AJ, Roberts H, et al. Trial design and reporting standards for intra-arterial cerebral thrombolysis for acute ischemic stroke. *Stroke; a journal of cerebral circulation*; 34. Epub ahead of print 2003. DOI: 10.1161/01.str.0000082721.62796.09.

3. Wahlgren N, Ahmed N, Dávalos A, et al. Thrombolysis with alteplase for acute ischaemic stroke in the Safe Implementation of Thrombolysis in Stroke-Monitoring Study (SITS-MOST): an observational study. *Lancet* 2007; 369: 275–282.

4. Von Kummer R, Broderick JP, Campbell BCV, et al. The heidelberg bleeding classification: Classification of bleeding events after ischemic stroke and reperfusion therapy. *Stroke* 2015; 46: 2981–2986.
